# Randomised clinical trial: 3-year interim analysis results of the VISION trial to evaluate the long-term safety of vonoprazan as maintenance treatment in patients with erosive oesophagitis

**DOI:** 10.1186/s12876-023-02772-w

**Published:** 2023-05-01

**Authors:** Ken Haruma, Yoshikazu Kinoshita, Takashi Yao, Ryoji Kushima, Junichi Akiyama, Nobuo Aoyama, Tatsuhiro Kanoo, Kouji Miyata, Naomi Kusumoto, Naomi Uemura

**Affiliations:** 1 Department of General Internal Medicine 2, Kawasaki Medical School, General Medical Center, Okayama, Japan; 2 General Internal Medicine, Hyogo Prefectural Harima-Himeji General Medical Center, Himeji, Hyogo Japan; 3 Department of Human Pathology, Juntendo University Graduate School of Medicine, Tokyo, Japan; 4 Department of Clinical Laboratory Medicine, Shiga University of Medical Science Hospital, Otsu, Shiga Japan; 5 Department of Gastroenterology and Hepatology, National Center for Global Health and Medicine, Tokyo, Japan; 6 GI Endoscopy and IBD Center, Aoyama Medical Clinic, Kobe, Hyogo Japan; 7 Takeda Pharmaceutical Company Ltd, Tokyo, Japan; 8 Department of Gastroenterology and Hepatology, National Center for Global Health and Medicine, Kohnodai Hospital, Ichikawa, Chiba Japan

**Keywords:** GERD or GORD, Oesophagus, Acidity (oesophageal), Gastric cancer, Carcinoid, Gastritis, Endoscopy

## Abstract

**Background:**

VISION is a randomised, phase 4, open-label, parallel-group, multicentre study conducted in 33 centres in Japan. The aim of this study was to assess the long-term safety of vonoprazan for maintenance treatment of healed erosive oesophagitis versus lansoprazole.

**Methods:**

Patients with endoscopically diagnosed erosive oesophagitis were randomised 2:1 to once-daily vonoprazan 20 mg or lansoprazole 30 mg, for a 4- to 8-week healing phase. Patients with endoscopically confirmed healing entered a 260-week maintenance phase with a once-daily starting dose of vonoprazan 10 mg or lansoprazole 15 mg. Primary endpoint was change in gastric mucosal histopathology.

**Results:**

Of 208 patients (vonoprazan, *n* = 139; lansoprazole, *n* = 69) entering the healing phase, 202 entered the maintenance phase (vonoprazan, *n* = 135; lansoprazole, *n* = 67). At 3 years, 109 vonoprazan-treated and 58 lansoprazole-treated patients remained on treatment. Histopathological evaluation of gastric mucosa showed that hyperplasia of parietal, foveolar and G cells was more common with vonoprazan than lansoprazole at week 156 of the maintenance phase. There was no marked increase in the occurrence of parietal, foveolar and G cell hyperplasia among patients in the vonoprazan group from week 48 to week 156. Histopathological evaluation of the gastric mucosa also showed no neoplastic changes in either group. No new safety issues were identified.

**Conclusions:**

In this interim analysis of VISION, no new safety concerns were identified in Japanese patients with healed erosive oesophagitis receiving vonoprazan or lansoprazole as maintenance treatment for 3 years. (CT.gov identifier: NCT02679508; JapicCTI-163153; Japan Registry of Clinical Trials: jRCTs031180040).

**Supplementary Information:**

The online version contains supplementary material available at 10.1186/s12876-023-02772-w.

## Background

Vonoprazan is a potassium-competitive acid blocker which, unlike conventional PPIs, reversibly inhibits the enzyme H^+^/K^+^ ATPase independently of acid pH [1, 2]. In Japan, vonoprazan was marketed in 2015 for the treatment of erosive oesophagitis, treatment of gastric and duodenal ulcers, eradication of *Helicobacter pylori*, and prevention of the recurrence of low-dose aspirin- or nonsteroidal anti-inflammatory drug-related gastric and duodenal ulcer [3]. The efficacy and safety of vonoprazan have been demonstrated in patients with erosive oesophagitis [4], and also when administered as maintenance treatment in patients with healed erosive oesophagitis refractory to conventional PPIs [5, 6]. Furthermore, vonoprazan has demonstrated non-inferiority to the conventional PPI lansoprazole in patients with erosive oesophagitis [7–10].

Since long-term maintenance treatment is recommended for patients with erosive oesophagitis [11], it is imperative to establish the long-term safety of the therapeutic agent. As vonoprazan has been shown to have a more potent acid inhibitory effect than conventional PPIs both in vivo and in vitro [1, 12], greater concern has been raised about side effects than with conventional PPIs [3]. A number of side effects have been reported with strong inhibitors of gastric acid secretion, including PPIs, but the most problematic is their potential association with neoplastic lesions such as gastric cancer and gastric neuroendocrine tumours (NET) [13–18]. The strong inhibition of gastric acid secretion causes hypergastrinaemia, and gastrin is known to have a proliferative effect on the mucosa of the digestive tract [19–23], including the gastric mucosa [24]. Findings from several observational studies suggest that long-term use of PPIs is associated with an increased risk of developing gastric cancer [25–32]; therefore, additional well-designed prospective studies are warranted to confirm the potential role of PPIs in gastric cancer development according to gastric histology at baseline [33]. Vonoprazan is a potent inhibitor of gastric acid secretion, resulting in increased gastrin levels compared with conventional PPIs. Hypergastrinaemia is thought to be one of the pathogenic causes of hyperplastic polyps [34], but there have been no prospective studies of vonoprazan-induced hypergastrinaemia. In addition, endoscopic findings show that long-term treatment with PPIs is associated with a high incidence of lesions such as fundic gland polyps [35], hyperplastic polyps [36], cobblestone mucosa [37], multiple white flat elevated lesions [38], and black spots [39].

Although the safety and efficacy of vonoprazan as maintenance therapy have been previously reported in a 52-week study [8], studies that examine the longer-term safety of vonoprazan maintenance treatment are needed. The objective of the Vonoprazan study In patients with eroSIve oesophagitis to evaluate lONg-term safety (VISION) is to evaluate the long-term safety and efficacy of vonoprazan 10 mg or 20 mg in patients receiving maintenance treatment for recurrent/reactivated erosive oesophagitis, compared with lansoprazole. Patients in the ongoing VISION trial will receive treatment for up to 5 years, but as vonoprazan is being used more frequently in clinical practice, we are reporting the results of the prespecified 3-year interim analysis to provide information on the long-term safety of vonoprazan that may be of reassurance to practitioners.

## Methods

### Study design

VISION is a 5-year, randomised, open-label, parallel-group, multicentre, phase 4 study conducted across 33 specialised medical institutions (university hospitals, general hospitals, and clinics; Additional file 1: Supporting Table 1) in Japan that are experienced in conducting clinical trials in gastro-oesophageal reflux disease or reflux oesophagitis. The study design comprises a 4- to 8-week healing phase followed by a 260-week maintenance phase [40].

Patients with endoscopically confirmed erosive oesophagitis (LA Classification Grades A–D) at the start of treatment (week 0) were randomised in a 2:1 ratio to receive either vonoprazan 20 mg or lansoprazole 30 mg once daily for a healing phase of either 4 weeks (for patients with confirmed endoscopic healing of erosive oesophagitis at week 4) or 8 weeks (for patients with no confirmed endoscopic healing of erosive oesophagitis at week 4). Patients with endoscopically confirmed healed erosive oesophagitis at week 4 or 8 of the healing phase then entered a 260-week maintenance phase. Healed erosive oesophagitis was defined as the absence of an endoscopic mucosal break (Grade 0 according to severity classification of erosive oesophagitis). During the maintenance phase, patients in the vonoprazan group were administered a starting dose of vonoprazan 10 mg once daily, and patients in the lansoprazole group were administered a starting dose of lansoprazole 15 mg once daily, for up to 260 weeks (thus a total of up to 268 weeks of treatment). Vonoprazan and lansoprazole doses were increased to 20 mg and 30 mg, respectively, if initial doses were insufficient for maintenance treatment of erosive oesophagitis. Patients were randomised and allocated to treatment via a web registration system and were administered the study drugs by the principal investigator or investigator.

During the healing phase, visits were planned at weeks 0 and 4, and also at week 8 for patients with no endoscopic healing of erosive oesophagitis at week 4. During the maintenance phase, an initial visit took place on initiation of maintenance treatment followed by visits every 12 weeks up to week 108 and visits every 24 weeks up to week 228, with a final visit at week 260.

For a uniform evaluation process of endoscopic images, a standard operating procedure for the evaluation was established. A start-up meeting at each site and two seminars for all investigators were held to thoroughly inform the standard operating procedures. The delegated investigator at each study site conducted and evaluated the endoscopy. Throughout the study period, the same investigator was preferred to conduct and evaluate the endoscopy as far as possible for each patient.

This study is being and has been conducted in accordance with the Declaration of Helsinki Ethical Guidelines for Clinical Research, Clinical Trials Act (since 1 April 2018), and all applicable laws and regulations, including, without limitation, data privacy laws and conflict of interest guidelines. Before the enactment of the Clinical Trials Act, this study was conducted in accordance with the Ethical Guidelines on Biomedical Research Involving Human Subjects (the Ministry of Education, Culture, Sports, Science and Technology [MEXT] and the Ministry of Health, Labour and Welfare [MHLW], 22 December 2014; this guideline has since been renamed the Ethical Guidelines for Life Science and Medical Research Involving Human Subjects). Owing to the enforcement of the Clinical Trials Act in Japan on 1 April 2018, VISION was classified as a ‘Specified Clinical Trial’ on 21 November 2018. The transformation review was conducted at ‘Certified Review Board of National Center for Global Health and Medicine’ (CRB3180021) certified by MHLW, and after that the study was approved and registered under the trial ID jRCTs031180040. Prior to this classification, the study was reviewed by the Ethical Review Boards of each study site, and informed written consent was obtained from all study participants. All authors have checked the study data and reviewed and approved the final manuscript. The study has been registered with ClinicalTrials.gov (NCT02679508, registration date 10/02/2016), JapicCTI-163153, and the Japan Registry of Clinical Trials (jRCTs031180040).

### Study participants

At the start of the healing phase, eligible patients were aged ≥ 20 years, *H. pylori*-negative, with endoscopically confirmed erosive oesophagitis (Los Angeles Classification Grades A to D). Key exclusion criteria for the healing phase included: previous treatment with PPIs (including vonoprazan) within 4 weeks before the start of the healing phase, a history of *H. pylori* eradication, clinically apparent hepatic impairment, renal impairment or renal failure, history of PPI hypersensitivity or allergy, and presence of a malignant tumour.

Patients were eligible to enter the maintenance phase if they had endoscopically confirmed erosive oesophagitis healing (defined as Grade 0 according to severity classification of erosive oesophagitis, i.e. no mucous membrane disorder) on completion of the healing phase (week 4 or 8). Patients were excluded from the maintenance phase if they received a PPI other than vonoprazan or lansoprazole during the healing phase.

### Outcomes

The primary endpoint was histopathological evaluation of the gastric mucosa, specifically, the presence or absence of neoplastic/dysplastic alteration of epithelial cells, parietal cell protrusion/hyperplasia, foveolar hyperplasia, enterochromaffin-like-cell hyperplasia (endocrine cell micronest; ECM) [41], and G-cell hyperplasia.

Secondary efficacy endpoints were endoscopic erosive oesophagitis healing rate at the end of the healing phase and erosive oesophagitis recurrence rate (recurrence was defined as LA Classification Grades A to D during the maintenance phase). Secondary safety endpoints were incidence of adverse events (AEs), endoscopic findings (presence or absence of fundic gland polyp, hyperplastic polyp, cobblestone mucosa, multiple white flat elevated lesions, and black spots), histological evaluation of gastritis according to the Sydney classification (inflammation [mononuclear infiltration], activity [neutrophilic infiltration], atrophy, intestinal metaplasia, *H. pylori* and incidence of gastric polyp).

Additional endpoints included serum gastrin level, pepsinogen I and II levels, pepsinogen I/II ratio, and serum chromogranin A level.

### Schedule of assessments

At the start of the healing phase, patient demographics, medical history and prior drug treatment were assessed, and *H. pylori* urea breath (after initial endoscopy) and CYP2C19 genotype were tested. Endoscopy was scheduled at weeks 0, 4 and 8 of the healing phase, and at weeks 48, 108, 156, 204, and 260 of the maintenance phase. The gastric biopsy for histopathological evaluation of the gastric mucosa and of gastritis (undertaken centrally by two pathologists specialised in gastrointestinal histopathology) was scheduled at week 0 of the healing phase and at weeks 48, 108, 156, 204, and 260 of the maintenance phase. At week 0, the biopsy sample was collected from the fundic gland region of the mid-greater curvature of the stomach and vestibular curvature within 2 cm of the pyloric ring. At subsequent timepoints, samples were collected near the Week 0 biopsy site. The presence or absence of *H. pylori* was determined by Giemsa staining and the evaluation of endocrine cells was determined by immunostaining with chromogranin A. Fasting serum gastrin, physical examination, vital signs, laboratory tests, medication adherence, concomitant drugs, and AEs were assessed at every visit in the healing phase after week 0 and every visit from week 12 onwards in the maintenance phase. AEs of interest included diarrhoea, gastrointestinal infections, bone fracture and pneumonia. Measurement of fasting levels of serum pepsinogen I and II and serum chromogranin A levels was scheduled at weeks 0, 4 and 8 of the healing phase, and at weeks 24, 48, 108, 156, 204, and 260 of the maintenance phase. Serum samples were collected under fasted conditions (after at least 10 h of fasting) preferably at the same time of the day for each patient. Analysis was performed at a central laboratory test measurement institution.

### Statistical methods

Based on two previous vonoprazan clinical studies (NCT01456260 and NCT01456247) [42, 43], sample sizes of 130 and 65 participants were proposed in the vonoprazan and lansoprazole groups, respectively. These were calculated to ensure that previously reported dropout rates of vonoprazan were taken into account to allow data collection from at least 100, 50, and 30 participants in the first, third, and fifth years, respectively.

Here, we report the interim analysis at 3 years (week 156 of the maintenance phase). This interim analysis was prespecified in the study protocol to evaluate the effect of long-term administration of vonoprazan on gastric mucosal tissue. This interim analysis was not intended to provide a basis for any decision on whether to continue or terminate the study.

## Results

### Patient disposition

**Fig. 1:**
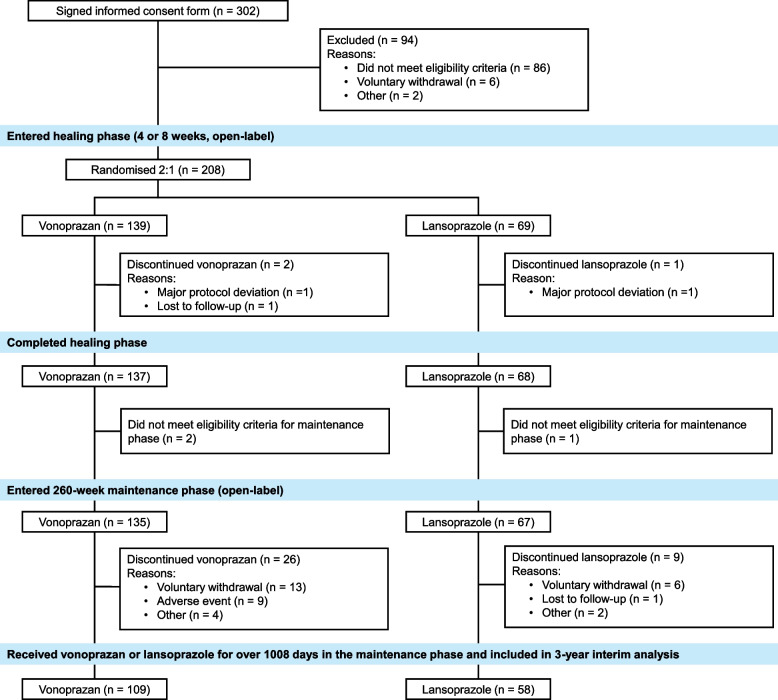
Patient disposition

The patient recruitment period was from March 2016 to February 2017. A total of 208 patients with endoscopically confirmed erosive oesophagitis were enrolled in the healing phase to receive vonoprazan 20 mg (*n* = 139) or lansoprazole 30 mg (*n* = 69; Fig. 1). In each of the two treatment groups, 97.1% of patients had endoscopically confirmed healed erosive oesophagitis at week 4 or 8 of the healing phase and entered the 260-week maintenance phase (vonoprazan, 135/139 [97.1%]; lansoprazole, 67/69 [97.1%]). The Safety Data Analysis Set in the maintenance phase comprised these 202 patients. Patient demographics and characteristics at baseline in the maintenance phase showed no marked differences between the vonoprazan and lansoprazole groups (Table 1).

**Table 1:** Patient demographics and baseline characteristics

Characteristic	VPZ (*N* = 135)	LPZ (*N* = 67)	Total (*N* = 202)
Age, years, mean (SD)	60.4 (11.81)	61.5 (12.16)	60.8 (11.91)
Male, n (%)	97 (71.9)	41 (61.2)	138 (68.3)
BMI at start of healing phase, kg/m^2^, mean (SD)	25.31 (3.72)	24.85 (3.33)	25.16 (3.59)
Consumption of alcohol, n (%)			
Every day	43 (31.9)	16 (23.9)	59 (29.2)
A few days a week	27 (20.0)	16 (23.9)	43 (21.3)
A few days a month	21 (15.6)	19 (28.4)	40 (19.8)
Never	44 (32.6)	16 (23.9)	60 (29.7)
Consumption of caffeine, n (%)	110 (81.5)	48 (71.6)	158 (78.2)
Endoscopy (oesophagus) at start of healing phase, n (%)			
Grade A	63 (46.7)	33 (49.3)	96 (47.5)
Grade B	49 (36.3)	25 (37.3)	74 (36.6)
Grade C	18 (13.3)	8 (11.9)	26 (12.9)
Grade D	5 (3.7)	1 (1.5)	6 (3.0)
CYP2C19 genotype test at start of healing phase, n (%)			
*1/*1	43 (31.9)	20 (29.9)	63 (31.2)
*1/*2	46 (34.1)	24 (35.8)	70 (34.7)
*1/*3	18 (13.3)	10 (14.9)	28 (13.9)
*2/*2	13 (9.6)	6 (9.0)	19 (9.4)
*2/*3	9 (6.7)	7 (10.4)	16 (7.9)
*3/*3	6 (4.4)	0 (0.0)	6 (3.0)
Serum gastrin level at start of healing phase, pg/mL			
Mean (SD)	130.2 (79.04)	155.4 (130.61)	138.6 (99.54)
< 200, n (%)	123 (91.1)	54 (80.6)	177 (87.6)
≥ 200, n (%)	12 (8.9)	13 (19.4)	25 (12.4)
Pepsinogen I/II ratio at start of healing phase			
Mean (SD)	5.45 (1.24)	5.24 (1.33)	5.38 (1.27)
> 2 to ≤ 3, n (%)	1 (0.7)	2 (3.0)	3 (1.5)
> 3, n (%)	134 (99.3)	65 (97.0)	199 (98.5)

*BMI* body mass index, *LPZ* lansoprazole, *SD* standard deviation, *VPZ* vonoprazan

Of the 202 patients who entered the maintenance phase, 167 (82.7%) completed over 1008 days of maintenance treatment (vonoprazan 10 or 20 mg, *n* = 109 [65.3%]; lansoprazole 15 or 30 mg, *n* = 58 [34.7%]; Fig. 1) and are included in the 3-year interim analysis.

### Histopathology of the gastric mucosa

**Fig. 2:**
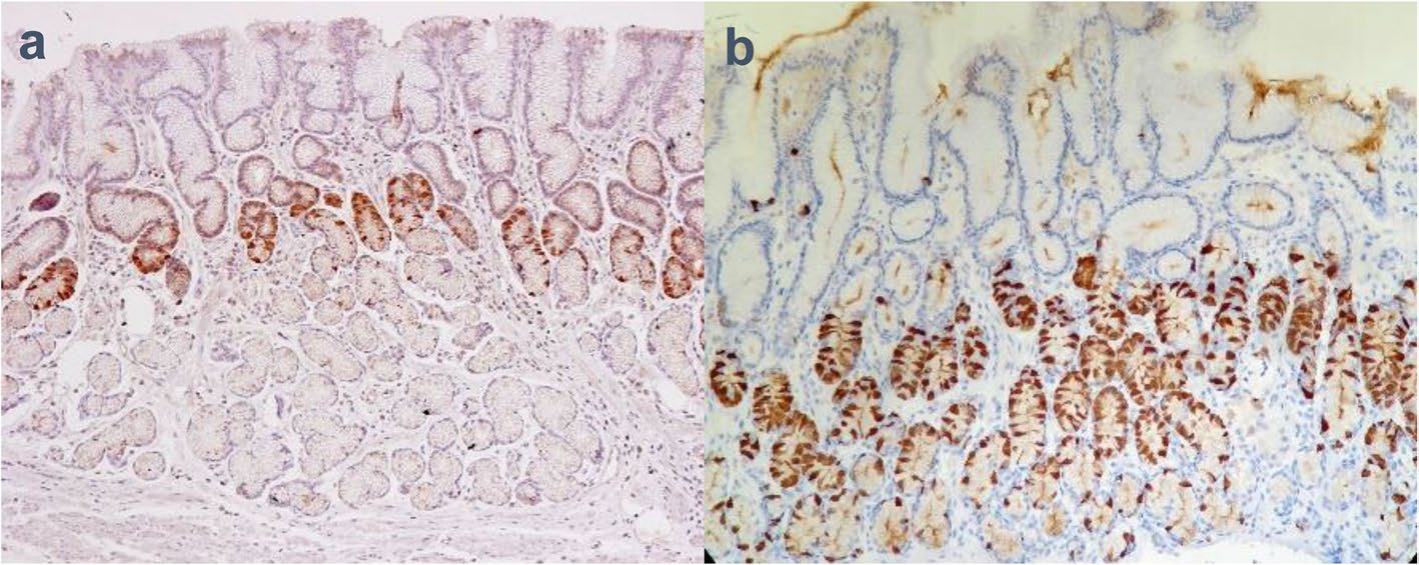
Immunohistological staining of G cells in the pyloric gland by gastrin: **a**) control; **b**) G cell hyperplasia in the vonoprazan group. The control sample of biopsy was from a normal gastric mucosa which was negative for *H. pylori* infection and did not have atrophy or inflammation

**Fig. 3:**
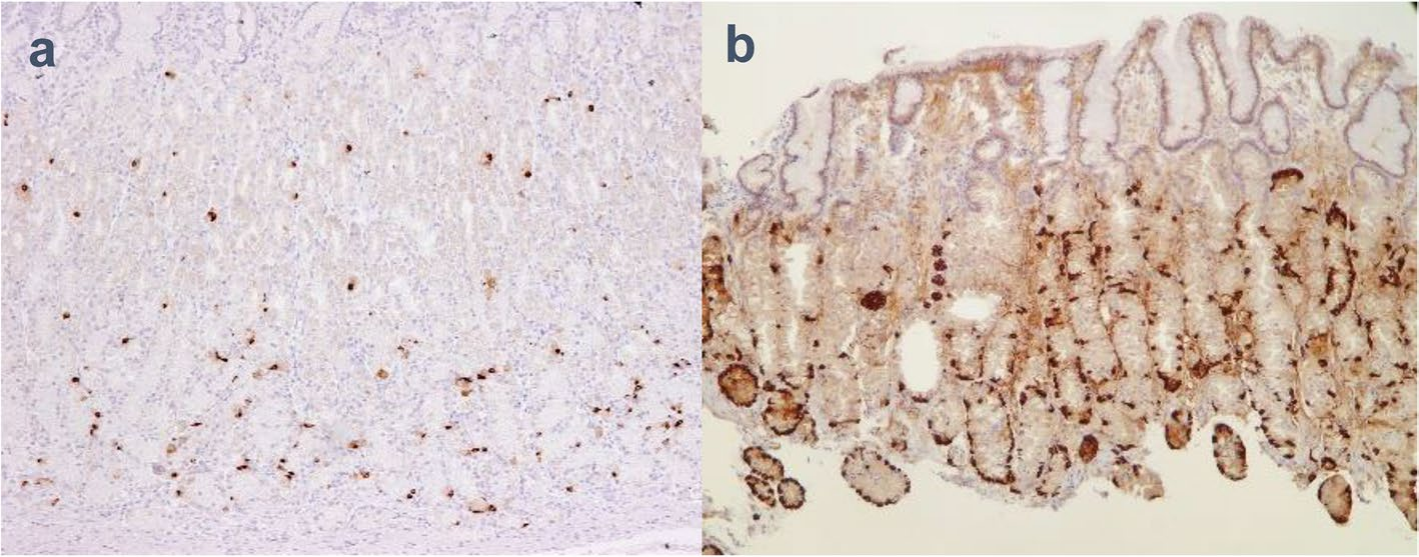
Immunohistological staining of the fundic gland by chromogranin A: **a**) control; **b**) enterochromaffin-like cell hyperplasia in the vonoprazan group. The control sample of biopsy was from a normal gastric mucosa which was negative for *H. pylori* infection and did not have atrophy or inflammation

No patient in either group showed neoplastic/dysplastic alteration of epithelial cells throughout the 156 weeks of treatment during the maintenance phase (Table 2). Hyperplasia of the parietal cells, foveolar cells, and G cells was more prominent among patients in the vonoprazan group than the lansoprazole group (Fig. 2; Table 2). After 156 weeks of maintenance treatment, parietal cell protrusion/hyperplasia was present in 102/109 (93.6%) vonoprazan-treated patients compared with 45/57 (78.9%) patients in the lansoprazole group. Inter-group differences for foveolar and G cell hyperplasia were marginally reduced at week 156, compared with weeks 48 and 108 (Table 2). There was no marked increase in the occurrence of parietal, foveolar, and G cell hyperplasia among patients in the vonoprazan group from week 48 to week 156. At week 156, one patient in the lansoprazole group developed atrophic ECM, while in the vonoprazan group one patient developed atrophic ECM and another developed hyperplastic ECM (Table 2; Fig. 3).

**Table 2:** Gastric mucosa histopathology at the start of the healing phase and at each visit during the maintenance phase up to week 156

**Histopathology**	**Findings, n (%)**	**VPZ**^a^	**LPZ**^b^
Neoplastic/dysplastic alteration of epithelial cells			
Start of healing phase	No	132 (98.5)	67 (100.0)
	Yes	0 (0.0)	0 (0.0)
	Unevaluable	2 (1.5)	0 (0.0)
Week 48	No	117 (95.1)	60 (95.2)
	Yes	0 (0.0)	0 (0.0)
	Unevaluable	6 (4.9)	3 (4.8)
Week 108	No	113 (98.3)	60 (100.0)
	Yes	0 (0.0)	0 (0.0)
	Unevaluable	2 (1.7)	0 (0.0)
Week 156	No	109 (100.0)	57 (100.0)
	Yes	0 (0.0)	0 (0.0)
	Unevaluable	0 (0.0)	0 (0.0)
Parietal cell protrusion/hyperplasia			
Start of healing phase	No	123 (91.8)	64 (95.5)
	Yes	10 (7.5)	3 (4.5)
	Unevaluable	1 (0.7)	0 (0.0)
Week 48	No	5 (4.1)	10 (15.9)
	Yes	115 (93.5)	53 (84.1)
	Unevaluable	3 (2.4)	0 (0.0)
Week 108	No	3 (2.6)	12 (20.0)
	Yes	111 (96.5)	48 (80.0)
	Unevaluable	1 (0.9)	0 (0.0)
Week 156	No	6 (5.5)	12 (21.1)
	Yes	102 (93.6)	45 (78.9)
	Unevaluable	1 (0.9)	0 (0.0)
Foveolar hyperplasia			
Start of healing phase	No	124 (92.5)	63 (94.0)
	Yes	6 (4.5)	4 (6.0)
	Unevaluable	4 (3.0)	0 (0.0)
Week 48	No	109 (88.6)	58 (92.1)
	Yes	6 (4.9)	1 (1.6)
	Unevaluable	8 (6.5)	4 (6.3)
Week 108	No	106 (92.2)	59 (98.3)
	Yes	6 (5.2)	1 (1.7)
	Unevaluable	3 (2.6)	0 (0.0)
Week 156	No	103 (94.5)	55 (96.5)
	Yes	5 (4.6)	2 (3.5)
	Unevaluable	1 (0.9)	0 (0.0)
G-cell hyperplasia^c^			
Start of healing phase	No	81 (60.4)	47 (70.1)
	Yes	44 (32.8)	17 (25.4)
	Unevaluable	9 (6.7)	3 (4.5)
Week 48	No	27 (22.0)	29 (46.0)
	Yes	90 (73.2)	32 (50.8)
	Unevaluable	6 (4.9)	2 (3.2)
Week 108	No	16 (13.9)	25 (41.7)
	Yes	91 (79.1)	31 (51.7)
	Unevaluable	8 (7.0)	4 (6.7)
Week 156	No	11 (10.1)	13 (22.8)
	Yes	93 (85.3)	40 (70.2)
	Unevaluable	5 (4.6)	4 (7.0)
ECL-cell hyperplasia, ECM			
Start of healing phase	No	133 (99.3)	67 (100.0)
	Yes	0 (0.0)	0 (0.0)
	Atrophic ECM^d^	0 (0.0)	0 (0.0)
	Hyperplastic ECM^e^	0 (0.0)	0 (0.0)
	Neoplastic ECM	0 (0.0)	0 (0.0)
	Typical carcinoid	0 (0.0)	0 (0.0)
	Unevaluable	1 (0.7)	0 (0.0)
Week 48	No	119 (96.7)	63 (100.0)
	Yes	1 (0.8)	0 (0.0)
	Atrophic ECM	0 (0.0)	0 (0.0)
	Hyperplastic ECM	1 (0.8)	0 (0.0)
	Neoplastic ECM	0 (0.0)	0 (0.0)
	Typical carcinoid	0 (0.0)	0 (0.0)
	Unevaluable	3 (2.4)	0 (0.0)
Week 108	No	111 (96.5)	60 (100.0)
	Yes	3 (2.6)	0 (0.0)
	Atrophic ECM	0 (0.0)	0 (0.0)
	Hyperplastic ECM	3 (2.6)	0 (0.0)
	Neoplastic ECM	0 (0.0)	0 (0.0)
	Typical carcinoid	0 (0.0)	0 (0.0)
	Unevaluable	1 (0.9)	0 (0.0)
Week 156	No	106 (97.2)	56 (98.2)
	Yes	2 (1.8)	1 (1.8)
	Atrophic ECM	1 (0.9)	1 (1.8)
	Hyperplastic ECM	1 (0.9)	0 (0.0)
	Neoplastic ECM	0 (0.0)	0 (0.0)
	Typical carcinoid	0 (0.0)	0 (0.0)
	Unevaluable	1 (0.9)	0 (0.0)

*ECL* enterochromaffin-like, *ECM* endocrine cell micronest, *LPZ* lansoprazole, *VPZ* vonoprazan

^a^Data based on 134 patients at the start of the healing phase; 123 patients at week 48, 115 patients at week 108 and 109 patients at week 156

^b^Data based on 67 patients at the start of the healing phase, 63 patients at week 48, 60 patients at week 108 and 57 patients at week 156

^c^G-cell hyperplasia was identified when gastrin-positive cells were continuously observed in a linear pattern [44]

^d^Atrophic ECM was defined as ECMs composed of < 10 endocrine cells, and regarded to be consistent with pseudohyperplasia [41]

^e^Hyperplastic ECM was defined as ECMs composed of > 10 endocrine cells and < 0.1 mm in diameter, and regarded to be 'micronodular and adenomatoid hyperplasia [41]

### Endoscopic findings in the stomach

At week 156, the percentage of patients with gastric polyps was marginally higher in the lansoprazole group (80.7%) than in the vonoprazan group (71.6%; Table 3). The proportion of patients with cobblestone mucosa was consistently higher in the vonoprazan group than in the lansoprazole group, from week 48 (9.8% vs 3.2%) to week 156 (14.7% vs 8.8%; Table 3). Multiple white flat elevated lesions were less common in the vonoprazan group than in the lansoprazole group at week 108 (3.5% vs 10.0%) and week 156 (6.4% vs 15.8%; Table 3). Black spots were also less common in the vonoprazan group than in the lansoprazole group at week 108 (4.3% vs 6.7%) and week 156 (5.5% vs 10.5%).

**Table 3:** Endoscopic findings of the stomach at the start of the healing phase and at each visit during maintenance phase up to week 156

**Endoscopic findings, n (%)**	**VPZ**^a^	**LPZ**^b^
Presence of fundic gland polyp		
Start of healing phase	59 (43.7)	29 (43.3)
Week 48	67 (54.5)	29 (46.8)
Week 108	78 (67.8)	39 (65.0)
Week 156	72 (66.1)	44 (77.2)
Presence of hyperplastic polyp		
Start of healing phase	5 (3.7)	0 (0.0)
Week 48	9 (7.3)	4 (6.5)
Week 108	13 (11.3)	6 (10.0)
Week 156	16 (14.7)	9 (15.8)
Cobblestone mucosa		
Start of healing phase	4 (3.0)	0 (0.0)
Week 48	12 (9.8)	2 (3.2)
Week 108	18 (15.7)	3 (5.0)
Week 156	16 (14.7)	5 (8.8)
Presence of multiple white flat elevated lesions		
Start of healing phase	3 (2.2)	5 (7.5)
Week 48	4 (3.3)	5 (8.1)
Week 108	4 (3.5)	6 (10.0)
Week 156	7 (6.4)	9 (15.8)
Presence of black spots		
Start of healing phase	1 (0.7)	0 (0.0)
Week 48	1 (0.8)	0 (0.0)
Week 108	5 (4.3)	4 (6.7)
Week 156	6 (5.5)	6 (10.5)
Incidence of gastric polyp		
Start of healing phase	63 (46.7)	29 (43.3)
Week 48	74 (60.2)	31 (49.2)
Week 108	83 (72.2)	40 (66.7)
Week 156	78 (71.6)	46 (80.7)

*ECM* endocrine cell micronest, *LPZ* lansoprazole, *VPZ* vonoprazan

^a^Data based on 135 patients at the start of the healing phase, 123 patients at week 48, 115 patients at week 108 and 109 patients at week 156

^b^Data based on 67 patients at the start of the healing phase, 62 patients at week 48, 60 patients at week 108 and 57 patients at week 156

### Histological evaluation of gastritis according to the Sydney classification

Incidences of the majority of gastritis parameters in the greater curvature of the antrum and middle gastric body were similar between vonoprazan and lansoprazole groups at week 156 (Table 4). Intestinal metaplasia in the greater curvature of the antrum were reported in 12 (9.0%) and 5 (7.5%) patients at baseline and in 6 (5.5%) and 5 (8.8%) patients at week 156 in the vonoprazan and lansoprazole groups, respectively. Two patients (1.5%) in the vonoprazan group and one (1.5%) in the lansoprazole group reported *H. pylori* in the greater curvature of the antrum at baseline. Of these three patients, the first patient from the vonoprazan group was found to have a small number of bacillus-like structures in the antrum, but no activity was seen in the evaluation of gastric biopsy tissue from the same site. Therefore, this case was determined as false positive, and the patient was enrolled in the study. The second patient from the vonoprazan group and a patient from the lansoprazole group showed identical histological findings of mild activity and moderate inflammation. Both cases were negative for urea breath test. The investigator was informed about the suspected mild *H. pylori* infection in both patients. The investigator ruled out *H. pylori* infection based on the endoscopic findings using the Kyoto classification of gastritis [45] in the patient from the vonoprazan group and therefore the patient was enrolled in the study. In the patient from the lansoprazole group, *H. pylori* infection could not be ruled out based on the Kyoto classification of gastritis and therefore this patient was not included in the study.

**Table 4:** Histological assessment of gastritis in the greater curvature of the antrum and middle gastric body at the start of the healing phase and at week 156 of the maintenance phase

**Histological assessment of gastritis parameter**	**Findings, n (%)**	**VPZ**^a^	**LPZ**^b^
Inflammation (mononuclear infiltration) in the greater curvature of the antrum			
Start of healing phase	No	103 (76.9)	49 (73.1)
	Yes	31 (23.1)	18 (26.9)
	Unevaluable	0	0
Week 156 of maintenance phase	No	93 (85.3)	50 (87.7)
	Yes	16 (14.7)	7 (12.3)
	Unevaluable	0	0
Inflammation (mononuclear infiltration) in the greater curvature of the middle gastric body			
Start of healing phase	No	85 (63.4)	37 (55.2)
	Yes	49 (36.6)	29 (43.3)
	Unevaluable	0	1 (1.5)
Week 156 of maintenance phase	No	83 (76.1)	52 (91.2)
	Yes	26 (23.9)	5 (8.8)
	Unevaluable	0	0
Activity (neutrophilic infiltration) in the greater curvature of the antrum			
Start of healing phase	No	133 (99.3)	65 (97.0)
	Yes	1 (0.7)	2 (3.0)
	Unevaluable	0	0
Week 156 of maintenance phase	No	109 (100.0)	57 (100.0)
	Yes	0	0
	Unevaluable	0	0
Activity (neutrophilic infiltration) in the greater curvature of middle gastric body			
Start of healing phase	No	133 (99.3)	66 (98.5)
	Yes	1 (0.7)	0
	Unevaluable	0	1 (1.5)
Week 156 of maintenance phase	No	109 (100.0)	57 (100.0)
	Yes	0	0
	Unevaluable	0	0
Atrophy in the greater curvature of the antrum			
Start of healing phase	No	71 (53.0)	40 (59.7)
	Yes	57 (42.5)	26 (38.8)
	Unevaluable	6 (4.5)	1 (1.5)
Week 156 of maintenance phase	No	91 (83.5)	46 (80.7)
	Yes	13 (11.9)	9 (15.8)
	Unevaluable	5 (4.6)	2 (3.5)
Atrophy in the greater curvature of the middle gastric body			
Start of healing phase	No	133 (99.3)	64 (95.5)
	Yes	1 (0.7)	2 (3.0)
	Unevaluable	0	1 (1.5)
Week 156 of maintenance phase	No	108 (99.1)	57 (100.0)
	Yes	0	0
	Unevaluable	1 (0.9)	0
Intestinal metaplasia in the greater curvature of the antrum			
Start of healing phase	No	122 (91.0)	62 (92.5)
	Yes	12 (9.0)	5 (7.5)
	Unevaluable	0	0
Week 156 of maintenance phase	No	103 (94.5)	52 (91.2)
	Yes	6 (5.5)	5 (8.8)
	Unevaluable	0	0
Intestinal metaplasia in the greater curvature of the middle gastric body			
Start of healing phase	No	134 (100.0)	66 (98.5)
	Yes	0	0
	Unevaluable	0	1 (1.5)
Week 156 of maintenance phase	No	109 (100.0)	56 (98.2)
	Yes	0	1 (1.8)
	Unevaluable	0	0
*H. pylori* in the greater curvature of the antrum			
Start of healing phase	No	132 (98.5)	66 (98.5)
	Yes	2 (1.5)	1 (1.5)
	Unevaluable	0	0
Week 156 of maintenance phase	No	109 (100.0)	57 (100.0)
	Yes	0	0
	Unevaluable	0	0
*H. pylori* in the greater curvature of the middle gastric body			
Start of healing phase	No	133 (99.3)	66 (98.5)
	Yes	0	0
	Unevaluable	1 (0.7)	1 (1.5)
Week 156 of maintenance phase	No	109 (100.0)	57 (100.0)
	Yes	0	0
	Unevaluable	0	0

*H. pylori Helicobacter pylori, LPZ* lansoprazole, *VPZ* vonoprazan

^a^Data based on 134 patients at the start of the healing phase and 109 patients at week 156 of the maintenance phase

^b^Data based on 67 patients at the start of the healing phase and 57 patients at week 156 of the maintenance phase

### Laboratory findings

**Fig. 4:**
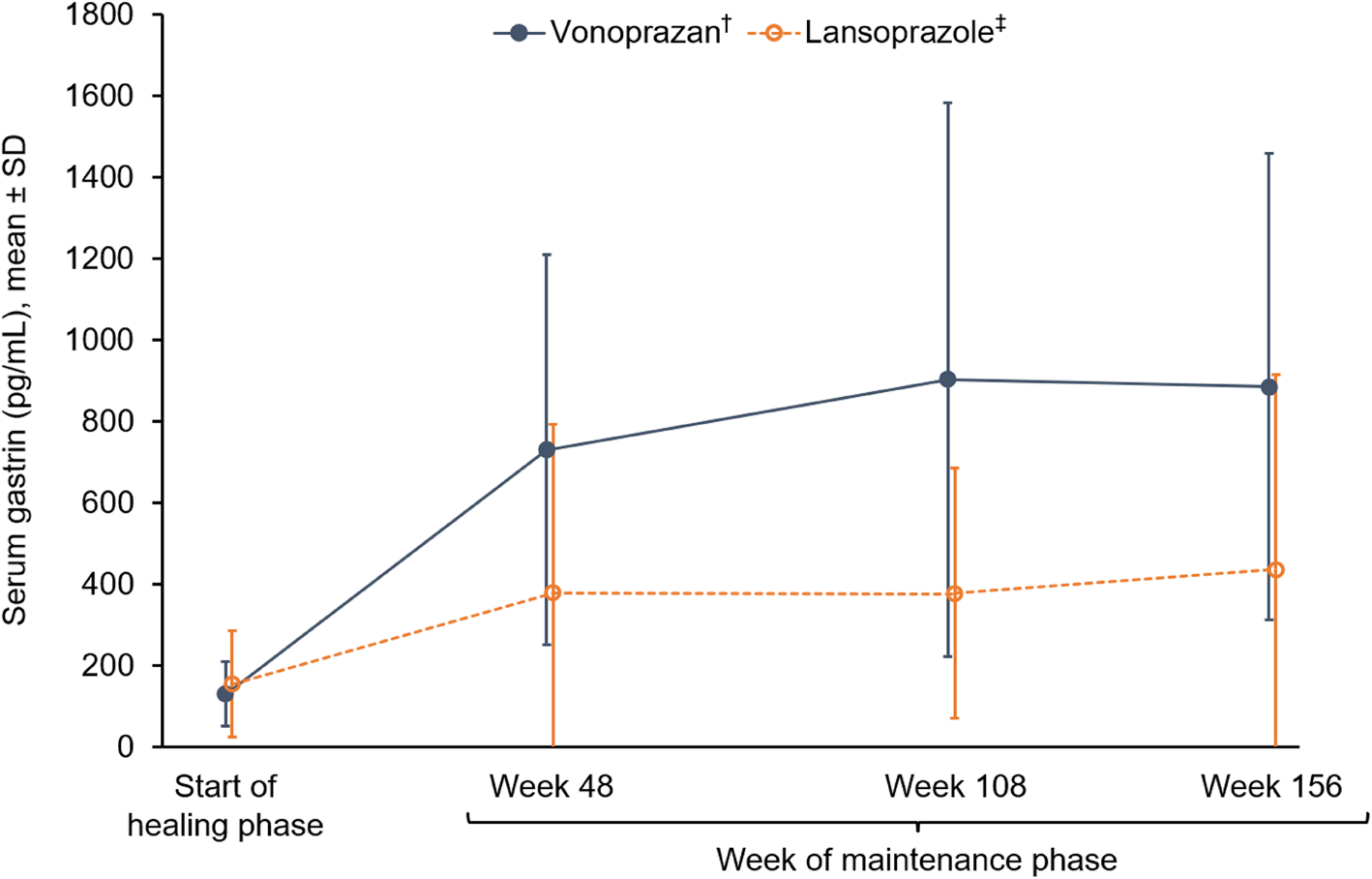
Serum gastrin levels at the start of the healing phase and during the maintenance phase up to week 156. ^†^Data based on 135 patients at the start of the healing phase, 124 patients at week 48, 115 patients at week 108 and 108 patients at week 156. ^‡^Data based on 67 patients at the start of the healing phase, 63 patients at week 48, 60 patients at week 108 and 57 patients at week 156. *SD* standard deviation

**Fig. 5:**
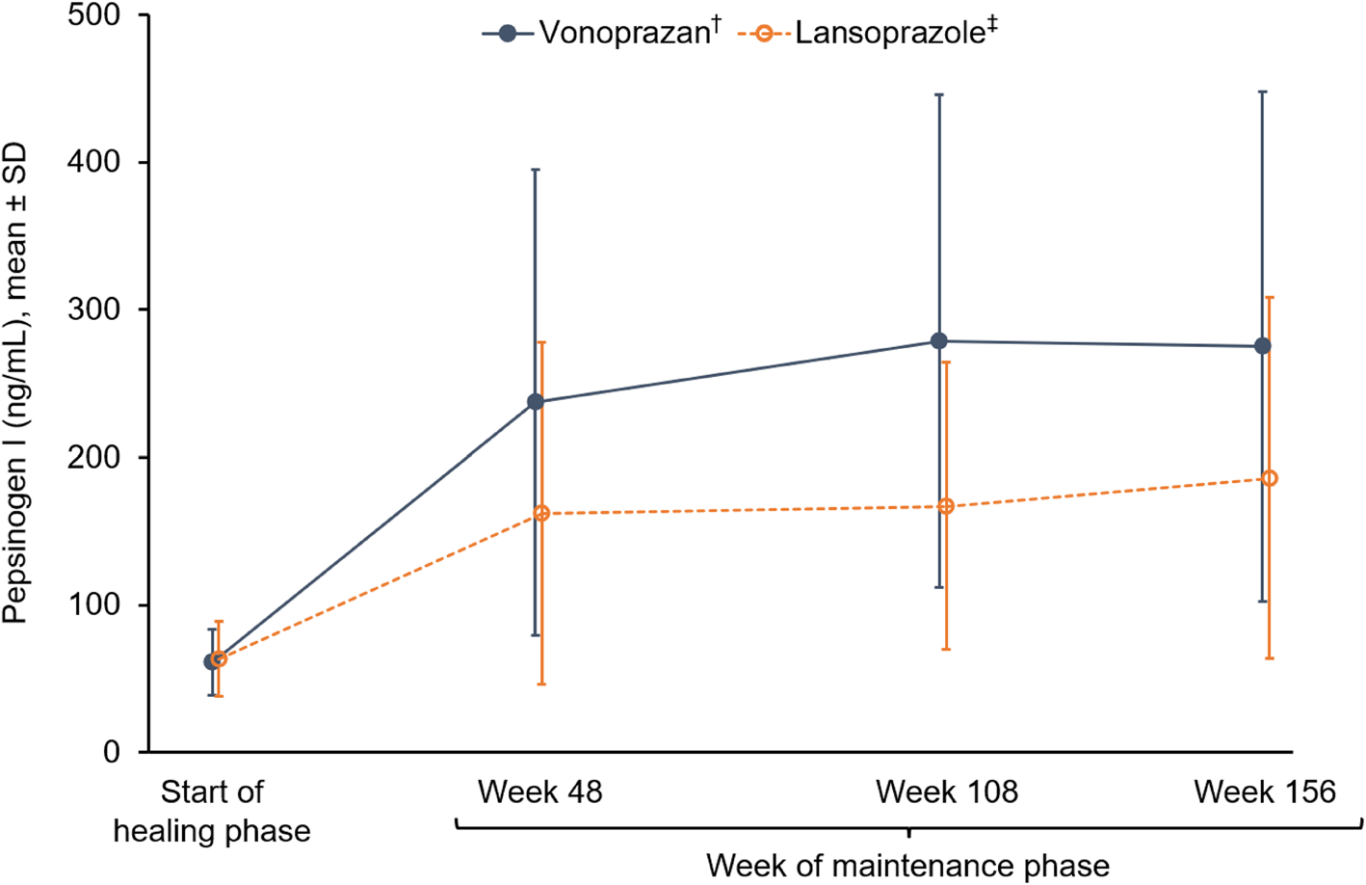
Pepsinogen I levels at the start of the healing phase and during the maintenance phase up to week 156. ^†^Data based on 135 patients at the start of the healing phase, 124 patients at week 48, 115 patients at week 108 and 108 patients at week 156. ^‡^Data based on 67 patients at the start of the healing phase, 63 patients at week 48, 60 patients at week 108 and 57 patients at week 156. *SD,* standard deviation

**Fig. 6:**
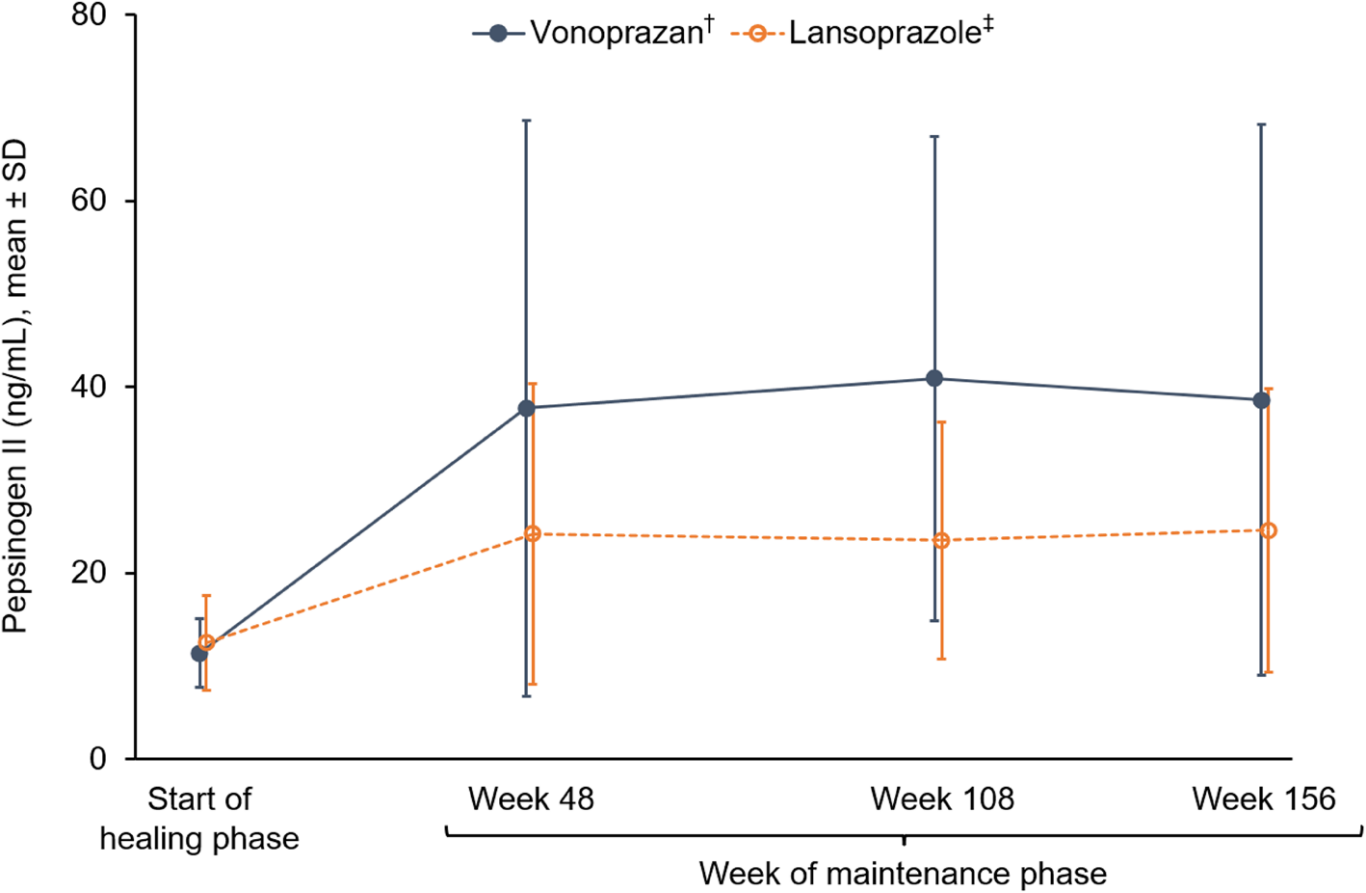
Pepsinogen II levels at the start of the healing phase and during the maintenance phase up to week 156. ^†^Data based on 135 patients at the start of the healing phase, 124 patients at week 48, 115 patients at week 108 and 108 patients at week 156. ^‡^Data based on 67 patients at the start of the healing phase, 63 patients at week 48, 60 patients at week 108 and 57 patients at week 156. *SD* standard deviation

**Fig. 7:**
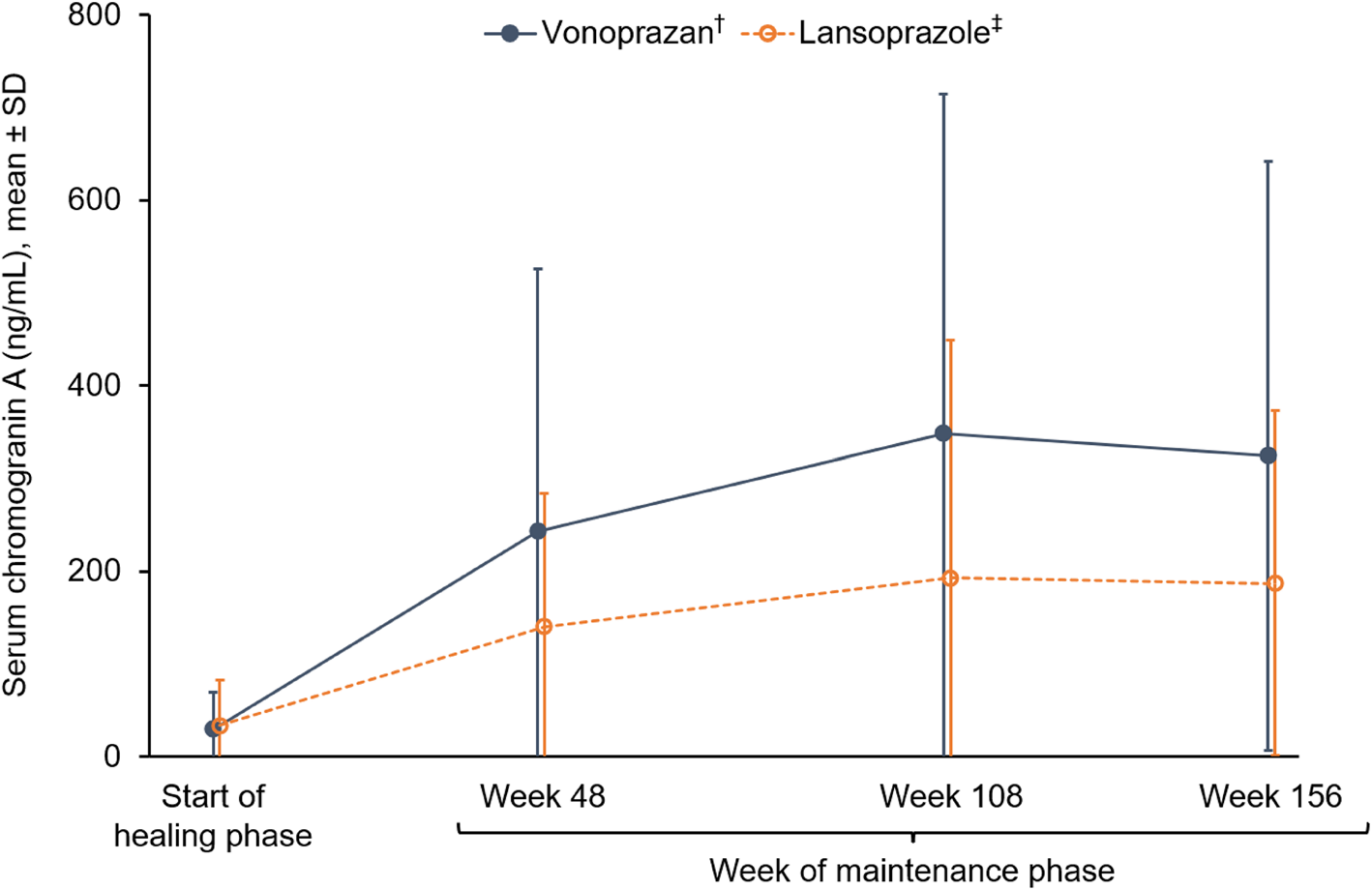
Serum chromogranin A levels at the start of the healing phase and during the maintenance phase up to week 156. ^†^Data based on 135 patients at the start of the healing phase, 124 patients at week 48, 115 patients at week 108 and 108 patients at week 156. ^‡^Data based on 67 patients at the start of the healing phase, 63 patients at week 48, 60 patients at week 108 and 57 patients at week 156. *SD* standard deviation

**Fig. 8:**
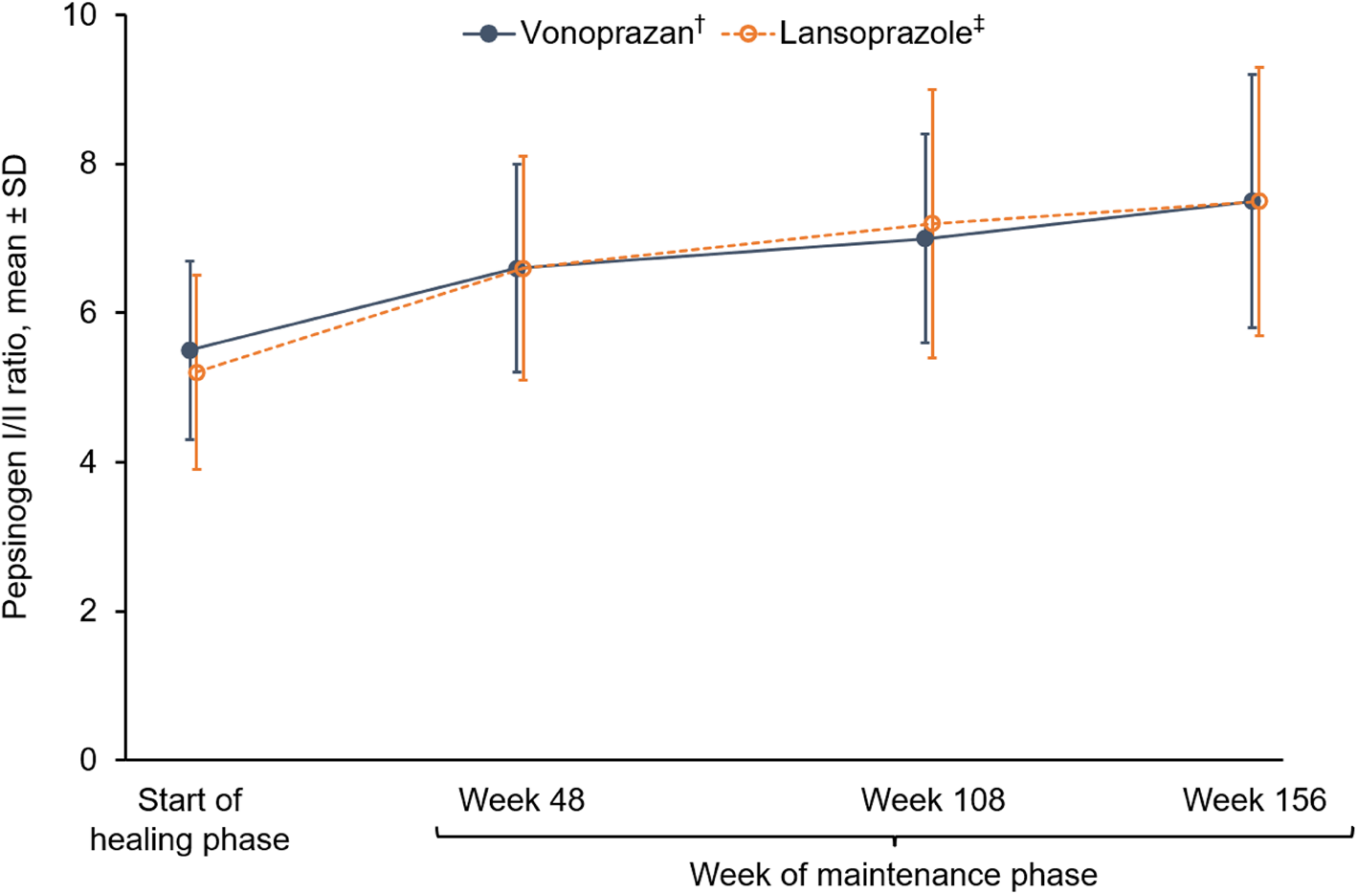
Pepsinogen I/II ratio at the start of the healing phase and during the maintenance phase up to week 156. ^†^Data based on 135 patients at the start of the healing phase, 124 patients at week 48, 115 patients at week 108 and 108 patients at week 156. ^‡^Data based on 67 patients at the start of the healing phase, 63 patients at week 48, 60 patients at week 108 and 57 patients at week 156. *SD* standard deviation

Up to week 156 of the maintenance phase, mean serum gastrin, pepsinogen I and II, and serum chromogranin A levels were consistently higher in the vonoprazan group than the lansoprazole group (Figs. 4, 5, 6 and 7). In the vonoprazan group, levels of serum gastrin, pepsinogen I and II, and serum chromogranin A progressively increased from the start of the healing phase to week 108 of the maintenance phase, but thereafter plateaued or decreased slightly, with no further increases observed between week 108 and week 156. Mean pepsinogen I/II ratios were similar for vonoprazan and lansoprazole groups from the start of the healing phase to week 156 of the maintenance phase (Fig. 8).

### Adverse events

AEs were reported in 89.6% of patients in the vonoprazan group and 95.5% of patients in the lansoprazole group (Table 5). AEs were mostly mild or moderate in severity, and the majority of AEs were not considered to be related to the vonoprazan or lansoprazole. During the maintenance phase up to week 156, 10 patients (7.4%) in the vonoprazan group and none in the lansoprazole group discontinued treatment because of an AE. No deaths were reported. The most commonly reported AEs, occurring in > 5% of the study population, were in the system organ classes ‘gastrointestinal disorders’ and ‘infections and infestations.’

**Table 5:** Overview of TEAEs in the maintenance phase up to week 156

TEAEs	VPZ (*N* = 135)	LPZ (*N* = 67)
Overview of TEAEs, n (%)		
All TEAEs	121 (89.6)	64 (95.5)
Related to study drug	54 (40.0)	31 (46.3)
Serious TEAEs	39 (28.9)	18 (26.9)
Related to study drug^a^	3 (2.2)	0 (0.0)
TEAEs leading to death	0 (0.0)	0 (0.0)
TEAEs occurring in > 5% of patients in any group, n (%)		
Gastrointestinal disorders	84 (62.2)	54 (80.6)
Gastric polyps	48 (35.6)	27 (40.3)
Gastric mucosal lesion	21 (15.6)	11 (16.4)
Gastritis erosive	11 (8.1)	8 (11.9)
Large intestine polyp	13 (9.6)	6 (9.0)
Diarrhoea	9 (6.7)	4 (6.0)
Gastrointestinal mucosal disorder	6 (4.4)	6 (9.0)
Constipation	7 (5.2)	2 (3.0)
Dyspepsia	2 (1.5)	4 (6.0)
Infections and infestations	80 (59.3)	38 (56.7)
Nasopharyngitis	44 (32.6)	26 (38.8)
Bronchitis	11 (8.1)	7 (10.4)
Influenza	12 (8.9)	1 (1.5)
Cystitis	8 (5.9)	2 (3.0)
Gastroenteritis	7 (5.2)	3 (4.5)
Pharyngitis	4 (3.0)	5 (7.5)
Herpes zoster	2 (1.5)	5 (7.5)
Tonsillitis	0	4 (6.0)
Musculoskeletal and connective tissue disorders	33 (24.4)	16 (23.9)
Back pain	7 (5.2)	3 (4.5)
Skin and subcutaneous tissue disorders	14 (10.4)	14 (20.9)
Eczema	7 (5.2)	6 (9.0)
Eye disorders	8 (5.9)	8 (11.9)
Cataract	2 (1.5)	5 (7.5)
Vascular and nervous system disorders	15 (11.1)	4 (6.0)
Hypertension	12 (8.9)	3 (4.5)
Dizziness	1 (0.7)	4 (6.0)

*TEAE* treatment-emergent adverse event, *LPZ* lansoprazole, *VPZ* vonoprazan

^a^Among AEs with incidences of < 5%, one case of gastric cancer, i.e. foveolar-type adenoma, was reported

Out of the 10 patients who discontinued due to an AE, the most common AEs were in the system organ classes ‘gastrointestinal disorders’, ‘neoplasms benign, malignant and unspecified (including cysts and polyps)’ and ‘infections and infestations.’ The study drug was withdrawn in two patients because of treatment-related AEs. One patient experienced a mild treatment-related AE (gastric mucosal lesion) and a moderate serious AE (SAE) (acute cholangitis). After the SAE, the study drug was withdrawn in view of future interruptions in the study procedures. A second patient experienced two treatment-related SAEs of mild intensity: abnormal hepatic function and leukopenia, and two mild SAEs not related to the treatment: tinnitus and loss of consciousness. The study drug was withdrawn due to the treatment-related SAEs and patient’s anxiety regarding these SAEs. In the remaining eight patients, the study drug was withdrawn due to non-treatment related AEs. The study drug was discontinued in four patients experiencing SAEs: suspected cancer of the tail of the pancreas in first patient, duodenal obstruction because of uncinate pancreas cancer in the second patient, right lung cancer in the third patient, and myelodysplastic syndrome in the fourth patient. Study drug was also discontinued in two patients experiencing mild AEs: abnormal hepatic function in one and generalised pain in the second patient, and one patient experiencing moderate AE of early colon cancer. In one patient the drug was withdrawn because of an SAE – a malignant tumour – presumed to have existed before study participation (Fig. 1) and therefore this patient was removed from the group of patients discontinuing study due to AEs.

Foveolar-type adenoma was reported as a serious AE (and not as part of the primary endpoint of histopathological evaluation showing presence or absence of neoplastic/dysplastic alteration of epithelial cells) in one patient at the scheduled week 156 visit. This patient had received 156 weeks of maintenance therapy with vonoprazan at the dosage of 10 mg/day. Endoscopy revealed an approximately 5-mm-sized reddish polyp with a raspberry-like morphology in the anterior wall of the upper body of the stomach. The diagnosis of foveolar-type adenoma was made following biopsy. When endoscopy was performed in routine clinical practice 8 weeks later, this lesion had disappeared due to the biopsy, but another reddish polyp, approximately 4 mm in size and with a morphology similar to that of the first lesion, was found at the posterior wall of the middle body of the stomach and was also diagnosed as foveolar-type adenoma based on a biopsy. At endoscopy in routine clinical practice 5 weeks later, the second lesion had also disappeared, following the biopsy. This patient’s serum gastrin level was in the normal range before the start of vonoprazan treatment (151 pg/mL) but increased to 662 pg/mL 4 weeks after starting vonoprazan 20 mg/day during the healing phase and remained elevated during maintenance therapy with vonoprazan 10 mg/day. Full details of this patient have been published in a case report [46].

## Discussion

This 3-year interim analysis of the VISION study shows that although vonoprazan treatment resulted in an increase in serum gastrin and chromogranin levels compared with the PPI lansoprazole, no neoplastic changes in epithelial mucosa or enterochromaffin-like cells were observed following long-term maintenance treatment with vonoprazan in patients with healed erosive oesophagitis. Although gastric NETs have been reported to occur in the setting of PPI-induced hypergastrinaemia [13–18], no gastric NETs were observed in either the vonoprazan or lansoprazole groups at 3 years in the current prospective study.

Serum chromogranin A level is known to be a marker for endocrine tumours [47], and in this study it was higher in the vonoprazan group than in the lansoprazole group. At week 156 of the maintenance phase, hyperplastic ECM was observed in one patient in the vonoprazan group and no gastric NETs were observed, but it is assumed that there is an increase in enterochromaffin-like cells throughout the gastric fundic gland mucosa, which requires careful observation in the future.

G-cell hyperplasia was observed in 32.8% of patients in the vonoprazan group and 25.4% of patients in the lansoprazole group at the beginning of the study but was found in progressively more patients in both treatment groups over time. At week 156, the prevalence was 85.3% in the vonoprazan group and 70.2% in the lansoprazole group, which may explain the higher serum gastrin levels in the vonoprazan group compared with the lansoprazole group. Few studies have investigated the effect of antisecretory drugs on the number of gastrin-producing G cells [48, 49]. Nielsen et al. studied G-cell number after 8 weeks of treatment with cimetidine in patients with duodenal ulcer and reported that G-cell hyperplasia occurred after treatment [48]. In addition, Pashankar et al. reported a significant increase in G-cell number in paediatric patients with reflux oesophagitis up to 7 years after treatment with omeprazole [49]. This is the first report of the effect of vonoprazan on G-cell number.

Both the vonoprazan and lansoprazole groups showed increases in serum pepsinogen I and II over time. In particular, the increase was higher in the vonoprazan group. Pepsinogens I and II are secreted by the chief cells and mucous neck cells [50]. It has been indicated that receptors for gastrin exist in the chief cells [51, 52]; therefore, it is likely that hypergastrinaemia caused by gastric acid secretion inhibitors resulted in increased serum pepsinogen I and II levels. Although hypergastrinaemia has been implicated in the development of gastric hyperplastic polyps [34, 53], the prevalence of gastric hyperplastic polyps was 3.7% in the vonoprazan group vs 0% in the lansoprazole group at study entry, 7.3% vs 6.5% at week 48, 11.3% vs 10.0% at week 108, and 14.7% vs 15.8% at week 156. Despite this increased prevalence in both groups compared with the start of the healing phase, there were no cases where treatment was terminated because of an increase in the number or growth of hyperplastic polyps.

In considering the relationship between gastric acid secretion inhibitors and gastric cancer, it has been pointed out that gastric acid secretion inhibitors enhance gastritis [54], resulting in the development of gastric mucosal atrophy and, in addition, intestinal metaplasia [55–59]. Although there are pros and cons to both sides of the argument [60, 61], as this is important in considering gastric carcinogenesis, in this prospective study we evaluated gastric biopsy tissue. Interestingly, in this study, improvement in inflammation and atrophy was observed in both vonoprazan and lansoprazole groups. Although normally there is no inflammation or atrophy of the gastric mucosa in *H. pylori*-uninfected subjects [62], gastric mucosal inflammation was observed at the start of treatment in 23.1% of antrum and 36.6% of corpus in the vonoprazan group and 26.9% of antrum and 43.3% of corpus in the lansoprazole group. In addition, at the start of the treatment, gastric biopsies showed atrophy of the antrum in 42.5% and 38.8% of patients in the vonoprazan and lansoprazole groups, respectively. *H. pylori* infection is the primary cause of gastric mucosal atrophy; therefore, at study entry, an *H. pylori* negative status by breath test was confirmed in all patients. The history of no eradication was confirmed by interview in all patients, but it is possible that patients with eradication history could have been included by chance. Further, atrophy of the gastric mucosa, particularly of the antral mucosa, may be caused not only by *H. pylori* infection but also by bile acid due to gastric reflux of duodenal fluid [63, 64]. At week 156, there was improvement in both the antrum and corpus in both groups, with the trend being more pronounced in the lansoprazole group. The reason for the improvement in inflammation is not clear. Haber et al. evaluated gastric mucosal inflammation before and after 6 years of treatment with lansoprazole in patients with reflux oesophagitis and found that inflammation of both antrum and corpus improved after 6 years in both *H. pylori*-positive and -negative patients [65]. It has been reported that lansoprazole has an anti-inflammatory effect, which may partly be responsible for the improvement in gastritis [65, 66]. As for atrophy of the gastric mucosa, reflux oesophagitis occurs more frequently in patients who are originally negative for *H. pylori* infection and have less atrophic gastritis, especially in corpus [67]. Among the patients enrolled in this study, atrophy of corpus at the beginning of the study was observed in one patient (0.7%) in the vonoprazan group and two patients (3.0%) in the lansoprazole group, and progression was not found during 156 weeks of maintenance treatment. On the other hand, atrophy of antrum was present in 42.5% of patients in the vonoprazan group and 38.8% of those in the lansoprazole group at the start of the study; these percentages improved to 11.9% and 15.8%, respectively, at week 156. The reason for this is not clear, but at week 156 there was an improvement in antrum in both groups. We speculate that the improvement of inflammation of the gastric mucosa might have contributed to the improved antrum. There was no occurrence or increase in intestinal metaplasia in either group.

For endoscopic findings other than hyperplastic polyps, fundic gland polyps were found in 72 (66.1%) patients in the vonoprazan group and 44 (77.2%) patients in the lansoprazole group at week 156; cobblestone mucosa in 16 (14.7%) and 5 (8.8%) patients, respectively; and multiple white flat elevated lesions in 7 (6.4%) and 9 (15.8%) patients, respectively. The incidence of gastric polypoid lesions increased with increasing duration of treatment. Fundic gland polyps were the most common type, but these were already present at entry to the maintenance phase in 43.7% of patients in the vonoprazan group and 43.3% in the lansoprazole group. The incidence of fundic polyps increases with the dose and duration of PPIs [35], and the results of this study are consistent with previous reports. No patients discontinued medication because of increased or enlarged gastric polyps, but one patient discontinued vonoprazan treatment at week 156 because of the presence of a foveolar-type adenoma. Recent studies have found that foveolar-type adenoma is a common type of gastric neoplastic lesion found in *H. pylori-*uninfected individuals [68, 69]. This type of lesion has been found both in individuals who have serum gastrin levels within the normal range and in PPI users who have elevated levels of serum gastrin [68, 69], and the tumorigenic mechanisms that result in the development of this type of lesion in *H. pylori*-uninfected individuals are currently unclear [46].

There was no difference in overall TEAEs between the vonoprazan and lansoprazole groups, but the proportion of patients who discontinued medication because of an AE was greater in the vonoprazan group than in the lansoprazole group.

A limitation of the study is the possibility of a false negative urea breath test. Another limitation of this study is that, although the site and method of histopathological sample collection was defined in the study protocol and procedures manual together with reference images, sampling was up to the endoscopist at each institution and could therefore be variable. However, histopathological evaluation of gastric mucosa and histological evaluation of gastritis were undertaken centrally in a blinded manner by two expert pathologists specialising in gastrointestinal histopathology, who also instructed the principal investigator on the method of sample collection before the start of the study. In addition, if there was any difference in the histopathological evaluation between these two pathologists, evaluation was determined after separate consultation with two other pathologists.

## Conclusions

In this 3-year interim analysis comparing the long-term safety of vonoprazan and lansoprazole as maintenance therapy in patients with healed erosive oesophagitis, pathological examination of gastric biopsy specimens showed no neoplastic/dysplastic alteration of epithelial cells, no gastric NETs, no exacerbation of gastritis, no development of atrophy, and no intestinal metaplasia. Approximately 80% of enrolled patients received treatment with vonoprazan or lansoprazole for at least 3 years. A further interim analysis is planned at week 204, with the final analysis occurring at week 260.

## Supplementary Information

**Additional file 1: Supporting Table 1.** List of principal investigators involved in the study.

## Data Availability

The datasets used and/or analysed during the current study are available from the corresponding author on reasonable request and will be provided after de-identification.

## Abbreviations

AE: Adverse event
CYP2C19: Cytochrome P450 2C19
ECM: Endocrine cell micronest
LA: Los Angeles
LPZ: Lansoprazole
NET: Neuroendocrine tumour
PPI: Proton pump inhibitor
SD: Standard deviation
TEAE: Treatment-emergent adverse event
VPZ: Vonoprazan
