# Supplementary material for: Randomised clinical trial: 3-year interim analysis results of the VISION trial to evaluate the long-term safety of vonoprazan as maintenance treatment in patients with erosive oesophagitis
Source: BMC Gastroenterol. 2023 May 1;23:139. doi: 10.1186/s12876-023-02772-w (PMC10152792; doi:10.1186/s12876-023-02772-w)
Supplement: Supplementary file 1 — Additional file 1: Supporting Table 1. List of principal investigators involved in the study. [file 12876_2023_2772_MOESM1_ESM.docx]

Supporting Table 1. List of principal investigators involved in the study

| **Name** | **Affiliation** |
| --- | --- |
| Akiko Shiotani | Kawasaki Medical School Hospital |
| Akio Shirane | Shirane Clinic |
| Eikichi Ihara | Department of Medicine and Bioregulatory Science,  Graduate school of Medical Sciences, Kyushu University |
| Harufumi Oizumi | Oizumi Medical Clinic |
| Hironori Masuyama | Masuyama Gastrointestinal Clinic |
| Hiroshi Harada | Harada Clinic |
| Hiroshi Morinaga | Morinaga Ueno Clinic |
| Hiroto Miwa | Hyogo College of Medicine |
| Hisanori Abe  Keiko Matsuyama | Arita GI Hospital |
| Junichi Akiyama | National Center for Global Health and Medicine |
| Katsuhiro Mabe  Kimitoshi Kubo | National Hospital Organization, Hakodate National Hospital |
| Kazuhiko Inoue | Junpukai Long Life Hospital |
| Kei Matsuki | Matsuki GI Clinic |
| Keishi Kawakubo | Kawakubo Clinic |
| Keisuke Kohga | Kohga Hospital |
| Koichi Kurahara | Matsuyama Red-cross Hospital |
| Kouji Mori | Mori Clinic |
| Mariko Hojo | Juntendo University School of Medicine |
| Masae Banno | Banno Clinic |
| Masaomi Maeda | Hanabata Clinic |
| Mitsugi Yasuda  Mitsuyoshi Kobayashi | KKR Takamatsu Hospital |
| Mitsushige Sugimoto | Shiga University of Medical Science Hospital |
| Nobuo Aoyama | Aoyama Clinic |
| Norihisa Ishimura | Shimane University Hospital |
| Shigemi Nakajima | JCHO Shiga Hospital |
| Shiro Kimura | Kimura Shiro Clinic |
| Shuji Kochi | Chihaya Hospital |
| Takehiro Arai | Tokatsu-tsujinaka Hospital |
| Teruki Oki | Oki Clinic |
| Tomofumi Murakami | Shimokitazawa Tomo Clinic |
| Tomoyuki Koike | Division of Gastroenterology, Tohoku University Graduate School of medicine |
| Tomoyuki Yada | Kohnodai Hospital, National Center for Global Health and Medicine |
| Yasuhiko Gotoh | Shinbeppu Hospital |
